# Carotid Intima–Media Thickness and Atherogenic Indices in Idiopathic Pulmonary Fibrosis: Evidence of Subclinical Atherosclerosis

**DOI:** 10.3390/life16060988

**Published:** 2026-06-11

**Authors:** Aydin Balci, Yasar Inkaya, Serkan Sen

**Affiliations:** 1 Department of Pulmonology, Afyonkarahisar Health Sciences University, Afyonkarahisar 03340, Turkey; aydin.balci@afsu.edu.tr (A.B.); yasar.inkaya@afsu.edu.tr (Y.I.); 2 Department of Medical Laboratory Techniques, Ataturk Vocational School of Health Services, Afyonkarahisar Health Sciences University, Afyonkarahisar 03340, Turkey

**Keywords:** idiopathic pulmonary fibrosis, carotid intima–media thickness, atherogenic indices, subclinical atherosclerosis, cardiovascular risk, lipid profile, visual analog scale

## Abstract

Background: Idiopathic pulmonary fibrosis (IPF) is a progressive interstitial lung disease characterized by poor prognosis and accumulating evidence of systemic vascular involvement. Although cardiovascular comorbidities are recognized in IPF, the presence and extent of subclinical atherosclerosis are yet to be fully characterized. This study determined whether patients with IPF exhibit increased carotid intima–media thickness (CIMT) and altered atherogenic indices compared with healthy controls. Methods: This retrospective case–control study enrolled 117 patients with IPF diagnosed based on international guidelines and 104 age- and sex-matched healthy controls. All participants underwent comprehensive pulmonary function testing, the 6-min walk test (6MWT), laboratory evaluation (including lipid profiles), and bilateral carotid Doppler ultrasonography for CIMT measurement. Atherogenic indices, including the atherogenic coefficient, cholesterol ratio risk (CRR), and atherogenic index, were calculated. Dyspnea severity was evaluated using the visual analog scale (VAS). Results: Patients with IPF exhibited significantly higher CIMT (0.78 ± 0.12 mm vs. 0.68 ± 0.10 mm, *p* < 0.001) and CRR (4.12 ± 1.23 vs. 3.45 ± 0.98, *p* < 0.001) compared with controls. After adjustment for age, sex, cumulative smoking exposure expressed as pack-years, BMI, and controlled hypertension, IPF status remained independently associated with higher CIMT (adjusted β = 0.086 mm, 95% CI: 0.057–0.115; *p* < 0.001) and CRR (adjusted β = 0.482, 95% CI: 0.191–0.773; *p* = 0.001). Furthermore, patients with IPF had significantly lower HDL cholesterol levels and higher VLDL cholesterol levels. CIMT correlated negatively with 6MWT distance (r = −0.312, *p* = 0.001) and positively with VAS dyspnea scores (r = 0.287, *p* = 0.002). Conclusions: Patients with IPF showed higher CIMT and more unfavorable atherogenic profiles than healthy controls, and these associations persisted after adjustment for major vascular risk factors. The observed relationships between CIMT, functional capacity, and dyspnea severity suggest a potential association between IPF and subclinical cardiovascular involvement. Prospective studies are warranted to clarify the clinical relevance and prognostic implications of these findings.

## 1. Background

Idiopathic pulmonary fibrosis (IPF) is a chronic, progressive, and ultimately fatal interstitial lung disease characterized by irreversible fibrotic remodeling of the pulmonary parenchyma [1]. With a median survival of 3–5 years after diagnosis, IPF is considered as one of the most severe forms of interstitial pneumonia [2]. It predominantly affects individuals, particularly men, aged over 60 years, with incidence rates ranging from 2.8 to 19 per 100,000 population depending on geographic region and diagnostic criteria [3,4]. The pathogenesis of IPF involves repetitive alveolar epithelial injury, aberrant wound healing, excessive extracellular matrix deposition, and progressive architectural distortion of the lung parenchyma [5,6]. Although the primary manifestations of IPF are respiratory, accumulating evidence suggests that this disease may have systemic implications, particularly affecting the cardiovascular system [7,8].

Cardiovascular comorbidities are increasingly recognized as significant contributors to morbidity and mortality in patients with IPF [9,10]. Recent systematic reviews and meta-analyses have documented high comorbidity burden in IPF populations, with pooled prevalence estimates of 45% for systemic hypertension, 38% for hyperlipidemia, and 23% for vascular disorders [11,12]. These findings underscore the importance of comprehensive cardiovascular risk assessment in IPF management. Epidemiological studies have demonstrated that patients with IPF have a higher prevalence of coronary artery disease, heart failure, and arrhythmias compared with age-matched controls [13,14]. The mechanisms underlying this association remain incompletely understood but may involve shared pathophysiological pathways, including chronic inflammation, oxidative stress, endothelial dysfunction, and profibrotic signaling [15,16]. Furthermore, hypoxemia, a common feature of advanced IPF, may contribute to cardiovascular dysfunction through multiple mechanisms, including pulmonary hypertension, increased sympathetic activity, and metabolic derangements [17,18].

Subclinical atherosclerosis, defined as the presence of atherosclerotic changes in the arterial wall before the onset of clinical symptoms, represents an important intermediate phenotype in cardiovascular disease development [19]. Carotid intima–media thickness (CIMT), measured by high-resolution B-mode ultrasonography, is a well-validated, non-invasive marker of subclinical atherosclerosis and has been shown to predict future cardiovascular events independently of traditional risk factors [20,21]. Increased CIMT reflects arterial wall thickening due to intimal hyperplasia, smooth muscle cell proliferation, and lipid accumulation—processes that are central to atherogenesis [22]. In addition to CIMT, various atherogenic indices derived from lipid profiles, such as the atherogenic coefficient, cholesterol ratio risk (CRR), and atherogenic index, have been proposed as simple, cost-effective tools for cardiovascular risk stratification [23,24].

While coronary artery calcium (CAC) scoring on routine chest computed tomography (CT) has emerged as a valuable opportunistic screening tool for cardiovascular disease in IPF patients [11,12], CIMT assessment offers several complementary advantages. Unlike CAC, which reflects calcified plaque burden and requires radiation exposure, CIMT is a radiation-free ultrasound-based technique that can detect earlier stages of atherosclerotic remodeling before calcification occurs. CIMT provides real-time functional assessment of arterial wall thickness and is particularly suitable for serial monitoring and research settings where repeated radiation exposure should be minimized. Furthermore, CIMT is widely accessible, cost-effective, and has been extensively validated as an independent predictor of cardiovascular events across diverse populations. Thus, CIMT and CAC should be viewed as complementary rather than competing modalities, each offering unique advantages in cardiovascular risk assessment for IPF patients.

Despite the recognized association between IPF and cardiovascular disease, few studies have systematically evaluated subclinical atherosclerosis in this population. Most existing research has focused on clinically manifest cardiovascular events rather than early vascular changes that may precede symptom onset [7,8]. Moreover, the relationship between subclinical atherosclerosis markers and disease-specific parameters in IPF, such as pulmonary function, exercise capacity, and dyspnea severity, remains poorly characterized. Understanding these relationships may provide insights into the systemic nature of IPF and inform strategies for cardiovascular risk mitigation in this vulnerable population.

The 6-min walk test (6MWT) is a simple, standardized assessment of functional exercise capacity that has prognostic value in IPF [25]. Reduced 6MWT distance has been associated with increased mortality and disease progression in IPF patients [26]. Similarly, dyspnea severity, commonly assessed using the visual analog scale (VAS), is a key symptom that significantly impacts quality of life and correlates with disease severity [27]. Whether these functional and symptomatic parameters are associated with subclinical atherosclerosis in IPF has not been adequately explored.

Given the emerging evidence of systemic vascular involvement in IPF and the lack of comprehensive studies examining subclinical atherosclerosis in this population, this study aimed to determine whether patients with IPF exhibit increased CIMT and altered atherogenic indices compared with healthy controls. We further examined correlations between these vascular markers and clinical parameters, including pulmonary function, exercise capacity, and dyspnea severity.

## 2. Methods

### 2.1. Study Design and Population

This retrospective case–control study was conducted at the Department of Pulmonology, Afyonkarahisar Health Sciences University, Turkey, between January 2018 and December 2022. The study protocol was approved by the Institutional Ethics Committee (approval number: 2025-1), and all procedures were performed in accordance with the Declaration of Helsinki.

IPF Patient Selection: Patients were included if they met the following criteria: (1) diagnosis of IPF according to the 2018 American Thoracic Society/European Respiratory Society/Japanese Respiratory Society/Latin American Thoracic Society (ATS/ERS/JRS/ALAT) clinical practice guidelines [1]; (2) age ≥40 years; (3) availability of high-resolution computed tomography (HRCT) of the chest demonstrating a usual interstitial pneumonia (UIP) pattern; and (4) comprehensive clinical, laboratory, and imaging data. The diagnosis of IPF was established through multidisciplinary discussion involving pulmonologists, radiologists, and, when indicated, pathologists. Patients with surgical lung biopsy underwent histopathological confirmation of UIP pattern.

Exclusion Criteria: Patients were excluded if they had: (1) known cardiovascular disease, including coronary artery disease, cerebrovascular disease, peripheral arterial disease, or heart failure; (2) diabetes mellitus; (3) chronic kidney disease (estimated glomerular filtration rate < 60 mL/min/1.73 m^2^); (4) active malignancy; (5) chronic inflammatory or autoimmune diseases (e.g., rheumatoid arthritis, systemic lupus erythematosus, inflammatory bowel disease); (6) current use of lipid-lowering agents, antiplatelet drugs (other than low-dose aspirin), or immunosuppressive medications; (7) uncontrolled systemic hypertension (systolic blood pressure > 160 mmHg or diastolic blood pressure > 100 mmHg despite antihypertensive therapy); (8) acute exacerbation of IPF within the preceding 3 months; (9) inability to perform pulmonary function tests or the 6-min walk test; or (10) poor-quality carotid ultrasound images precluding accurate CIMT measurement. Patients with controlled hypertension (blood pressure < 140/90 mmHg on stable antihypertensive therapy) or untreated mild hyperlipidemia were included, and these comorbidities were systematically documented.

Control Group Selection: Healthy controls were recruited from individuals undergoing routine health examinations at our institution. Controls were matched to IPF patients for age (±5 years) and sex. Inclusion criteria for controls were: (1) absence of respiratory symptoms; (2) normal spirometry; (3) normal chest radiograph; and (4) absence of known chronic diseases. Controls were subject to the same exclusion criteria as IPF patients.

### 2.2. Clinical and Laboratory Assessments

Pulmonary Function Testing: Spirometry and diffusing capacity for carbon monoxide (DLCO) were performed according to ATS/ERS standards using a computerized spirometry system (Vmax Encore 229, CareFusion, San Diego, CA, USA) [28]. Measurements included forced vital capacity (FVC), forced expiratory volume in 1 s (FEV_1_), FEV_1_/FVC ratio, and DLCO. Results were expressed as percentages of predicted values based on reference equations.

6-min Walk Test: The 6MWT was performed according to ATS guidelines in a 30-m corridor [29]. Patients were instructed to walk as far as possible in 6 min, with standardized encouragement provided at 1-min intervals. The distance covered (6MWT distance) was recorded in meters. Oxygen saturation and heart rate were monitored continuously using pulse oximetry.

Dyspnea Assessment: Dyspnea severity was evaluated using a 100-mm visual analog scale (VAS), where 0 mm represented “no dyspnea” and 100 mm represented “worst imaginable dyspnea” [30]. Patients were asked to mark their current level of dyspnea on the scale.

Laboratory Measurements: Venous blood samples were collected after an overnight fast (≥12 h). Serum lipid profiles, including total cholesterol, triglycerides, high-density lipoprotein cholesterol (HDL-C), low-density lipoprotein cholesterol (LDL-C), and very-low-density lipoprotein cholesterol (VLDL-C), were measured using enzymatic colorimetric methods on an automated analyzer (Cobas 6000, Roche Diagnostics, Rotkreuz, Switzerland). LDL-C was calculated using the Friedewald equation when triglycerides were <400 mg/dL [31]. Additional biochemical parameters, including glucose, creatinine, and high-sensitivity C-reactive protein (hs-CRP), were also measured.

Atherogenic Indices: The following atherogenic indices were calculated from lipid profile data:

1. Atherogenic Coefficient (AC): AC = (Total Cholesterol − HDL-C)/HDL-C [32].
2. Cholesterol Ratio Risk (CRR): CRR = Total Cholesterol/HDL-C [33].
3. Atherogenic Index (AI): AI = log_10_(Triglycerides/HDL-C) [34].

### 2.3. Carotid Intima–Media Thickness Measurement

Bilateral carotid artery ultrasonography was performed using a high-resolution B-mode ultrasound system (Logiq P5, GE Healthcare, Chicago, IL, USA) equipped with a 7.5-MHz linear-array transducer. All examinations were conducted by experienced vascular sonographers (>5 years of experience) who were blinded to the clinical status (IPF vs. control) and laboratory results of the participants.

Patients were examined in the supine position with the neck slightly extended and rotated approximately 45 degrees away from the side being examined to optimize visualization of the carotid artery. The common carotid artery (CCA), carotid bulb, and internal carotid artery were systematically evaluated bilaterally.

CIMT was measured at the far wall of the distal CCA, approximately 10 mm proximal to the carotid bulb, in a plaque-free segment. The intima–media thickness was defined as the distance between the lumen–intima interface and the media–adventitia interface. Three measurements were obtained from each side (left and right CCA), and the mean of six measurements was calculated and used for analysis. All measurements were performed according to the Mannheim Consensus criteria for CIMT assessment [20,32].

CIMT was defined as the distance between the lumen–intima interface and the media–adventitia interface of the arterial wall [22]. An average CIMT >0.9 mm was considered abnormal, and the presence of atherosclerotic plaques (focal thickening >1.5 mm or >50% of the surrounding CIMT) was documented [35].

### 2.4. Statistical Analysis

Statistical analyses were performed using SPSS version 25.0 (IBM Corp., Armonk, NY, USA). Continuous variables were tested for normality using the Kolmogorov–Smirnov test and visual inspection of histograms and Q-Q plots. Normally distributed continuous variables were expressed as mean ± standard deviation (SD), and non-normally distributed variables were expressed as median (interquartile range, IQR). Categorical variables were expressed as frequencies and percentages.

Comparisons between IPF patients and controls were performed using independent samples *t*-tests for normally distributed continuous variables, Mann–Whitney U tests for non-normally distributed continuous variables, and chi-square tests or Fisher’s exact tests for categorical variables. To address confounding in the primary between-group comparisons, separate multivariable linear regression models were constructed with study group (IPF vs. control) as the main independent variable and CIMT (primary outcome), CRR, atherogenic coefficient (AC), and atherogenic index (AI) as dependent variables. The control group was used as the reference category. Covariates were selected a priori based on clinical relevance and reviewer concern regarding confounding: age, sex, cumulative smoking exposure, BMI, and controlled hypertension. Smoking exposure was handled primarily as pack-years because the groups differed substantially in former-smoking prevalence and cumulative exposure; a sensitivity approach using categorical smoking status (never/former/current) yielded directionally consistent results. Hypertension was included because controlled hypertension differed between groups and is an established determinant of CIMT. Effect estimates are reported as unstandardized β coefficients representing adjusted mean differences with 95% confidence intervals (CI) and *p*-values.

In addition, multiple linear regression analysis was performed within the IPF cohort to identify independent predictors of CIMT, with adjustment for potential confounders, including age, sex, smoking exposure (pack-years), body mass index (BMI), and relevant biochemical and clinical parameters. Variables with *p* < 0.10 in univariate analysis were entered into the multivariable model. Multicollinearity was assessed using variance inflation factors (VIF), with VIF >5 indicating significant multicollinearity.

A two-tailed *p*-value <0.05 was considered statistically significant for all analyses.

## 3. Results

### 3.1. Baseline Characteristics

The study population consisted of 117 patients with confirmed IPF and 104 healthy controls matched for age (±5 years) and sex. The baseline demographic, clinical, and laboratory characteristics of IPF patients and controls are presented in Table 1. The mean age was 66.4 ± 8.7 years in the IPF group and 65.8 ± 8.3 years in the control group (*p* = 0.612). The proportion of males was similar between groups (IPF: 71.8% vs. controls: 69.2%, *p* = 0.673). However, IPF patients had a significantly higher prevalence of former smoking (62.4% vs. 28.8%, *p* < 0.001) and greater cumulative smoking exposure (pack-years: 28.6 ± 22.4 vs. 12.3 ± 15.7, *p* < 0.001) compared with controls.

Regarding comorbidities, 34.2% of IPF patients had controlled systemic hypertension (on stable antihypertensive therapy with blood pressure <140/90 mmHg), compared with 18.3% of controls (*p* = 0.007). Untreated mild hyperlipidemia (total cholesterol 200–239 mg/dL without lipid-lowering therapy) was documented in 28.2% of IPF patients and 21.2% of controls (*p* = 0.224). Other documented vascular disorders (history of transient ischemic attack or peripheral arterial disease symptoms not meeting exclusion criteria) were present in 6.0% of IPF patients and 1.9% of controls (*p* = 0.134). These comorbidity prevalences are consistent with recent meta-analyses documenting high cardiovascular risk factor burden in IPF populations [11,12].

Body mass index (BMI) was slightly lower in IPF patients compared with controls (26.8 ± 4.2 kg/m^2^ vs. 28.1 ± 3.9 kg/m^2^, *p* = 0.021). Baseline biochemical parameters, including fasting glucose, creatinine, and hs-CRP, are also presented in Table 1.

### 3.2. Pulmonary Function and Functional Capacity

As expected, IPF patients exhibited significantly impaired pulmonary function compared with controls (Table 2). Mean FVC was 62.4 ± 14.8% predicted in IPF patients versus 98.6 ± 12.3% predicted in controls (*p* < 0.001). Similarly, DLCO was markedly reduced in IPF patients (48.7 ± 13.6% predicted vs. 94.2 ± 11.8% predicted, *p* < 0.001). The FEV_1_/FVC ratio was preserved in both groups, consistent with the restrictive physiology of IPF.

The 6MWT distance was significantly shorter in IPF patients compared with controls (348.6 ± 92.4 m vs. 486.3 ± 68.7 m, *p* < 0.001). VAS dyspnea scores were markedly higher in IPF patients (54.3 ± 18.6 mm vs. 8.2 ± 6.4 mm, *p* < 0.001).

### 3.3. Lipid Profiles and Atherogenic Indices

Lipid profiles and atherogenic indices are presented in Table 3. IPF patients had significantly lower HDL-C levels compared with controls (46.3 ± 11.2 mg/dL vs. 52.1 ± 10.8 mg/dL, *p* < 0.001) and higher VLDL-C levels (28.4 ± 9.6 mg/dL vs. 24.1 ± 7.8 mg/dL, *p* = 0.001). Total cholesterol, LDL-C, and triglyceride levels did not differ significantly between groups.

All three atherogenic indices were significantly elevated in IPF patients compared with controls. The atherogenic coefficient was 3.68 ± 1.18 in IPF patients versus 3.12 ± 0.94 in controls (*p* < 0.001). CRR was 4.12 ± 1.23 in IPF patients versus 3.45 ± 0.98 in controls (*p* < 0.001). The atherogenic index was 0.54 ± 0.22 in IPF patients versus 0.43 ± 0.18 in controls (*p* < 0.001).

### 3.4. Carotid Intima–Media Thickness

CIMT was significantly higher in IPF patients compared with controls (0.78 ± 0.12 mm vs. 0.68 ± 0.10 mm, *p* < 0.001) (Table 4). The proportion of patients with abnormal CIMT (>0.9 mm) was significantly higher in the IPF group (29.1% vs. 9.6%, *p* < 0.001). Atherosclerotic plaques were detected in 23.9% of IPF patients compared with 11.5% of controls (*p* = 0.015).

### 3.5. Correlations Between CIMT, Atherogenic Indices, and Clinical Parameters

Correlation analyses revealed several significant associations (Table 5). In IPF patients, CIMT correlated negatively with 6MWT distance (r = −0.312, *p* = 0.001) and positively with VAS dyspnea scores (r = 0.287, *p* = 0.002). CIMT also correlated negatively with FVC% predicted (r = −0.246, *p* = 0.008) and DLCO% predicted (r = −0.268, *p* = 0.004).

Among atherogenic indices, CRR showed the strongest correlations with CIMT (r = 0.342, *p* < 0.001). The atherogenic coefficient and atherogenic index also correlated significantly with CIMT (r = 0.318, *p* < 0.001 and r = 0.294, *p* = 0.001, respectively).

VAS dyspnea scores exhibited significant correlations with multiple parameters, including FVC% predicted (r = −0.524, *p* < 0.001), DLCO% predicted (r = −0.486, *p* < 0.001), 6MWT distance (r = −0.612, *p* < 0.001), and hs-CRP (r = 0.378, *p* < 0.001).

### 3.6. Subgroup Analysis by Smoking Status

To address potential confounding by smoking, we performed subgroup analyses stratified by smoking status (Table 6). Among former smokers, IPF patients had significantly higher CIMT compared with controls (0.80 ± 0.13 mm vs. 0.72 ± 0.11 mm, *p* = 0.006). Similarly, among never-smokers, IPF patients exhibited higher CIMT than controls (0.74 ± 0.10 mm vs. 0.66 ± 0.09 mm, *p* = 0.002). These findings suggest that the association between IPF and increased CIMT persists across smoking strata.

### 3.7. Adjusted Between-Group Comparisons

To further address potential confounding in the primary IPF versus control comparisons, multivariable linear regression analyses were performed with group status as the main predictor. After adjustment for age, sex, smoking exposure expressed as pack-years, BMI, and controlled hypertension, IPF status remained independently associated with higher CIMT. Similarly, IPF status was independently associated with higher CRR, AC, and AI after covariate adjustment. These findings indicate that the observed between-group differences in CIMT and atherogenic indices were not fully explained by differences in age, sex distribution, smoking exposure, BMI, or controlled hypertension (Table 7).

### 3.8. Multiple Linear Regression Analysis

In addition, multiple linear regression analysis was performed within the IPF cohort to identify independent predictors of CIMT, with adjustment for potential confounders, including age, sex, smoking exposure (pack-years), body mass index (BMI), and relevant biochemical and clinical parameters. Variables with *p* < 0.10 in univariate analysis were entered into the multivariable model. Multicollinearity was assessed using variance inflation factors (VIF), with VIF >5 indicating significant multicollinearity. The results of the multivariable regression model identifying independent predictors of CIMT are presented in Table 8.

## 4. Discussion

This study demonstrates that patients with IPF exhibit significantly elevated CIMT and unfavorable atherogenic profiles compared with age- and sex-matched healthy controls, suggesting enhanced subclinical atherosclerosis in this population. These findings are consistent with recent systematic reviews and meta-analyses documenting high cardiovascular comorbidity burden in IPF [11,12]. Walters et al. (2025) [12] reported pooled prevalence estimates of 45% for systemic hypertension, 38% for hyperlipidemia, and 23% for vascular disorders in IPF populations, underscoring the importance of comprehensive cardiovascular risk assessment in these patients. Our study extends this literature by providing detailed characterization of subclinical atherosclerotic changes using CIMT and atherogenic indices, and by examining their relationships with disease-specific functional parameters.

The observed elevation in CIMT among IPF patients is consistent with emerging evidence of systemic vascular involvement in this disease. While coronary artery calcium (CAC) scoring on routine chest CT has been proposed as an opportunistic screening tool for cardiovascular disease in IPF [11,12], our findings support the complementary value of CIMT assessment. Unlike CAC, which reflects calcified plaque burden and requires radiation exposure, CIMT provides a radiation-free, ultrasound-based assessment that can detect earlier stages of atherosclerotic remodeling before calcification occurs. This is particularly relevant for IPF patients who already undergo frequent chest CT scans for disease monitoring. Furthermore, CIMT is widely accessible, cost-effective, and suitable for serial monitoring in both clinical and research settings. Thus, CIMT and CAC should be viewed as complementary modalities, each offering unique advantages in cardiovascular risk stratification for IPF patients.

Importantly, the adjusted between-group analyses showed that IPF status remained independently associated with CIMT and key atherogenic indices even after adjustment for age, sex, smoking exposure, BMI, and controlled hypertension. This finding strengthens the robustness of the primary comparison by suggesting that the observed vascular and lipid-related differences between IPF patients and controls were not solely attributable to traditional cardiovascular risk factors. Nevertheless, because of the retrospective and cross-sectional design, these results should be interpreted as associative rather than causal.

Our cohort’s comorbidity profile—with 34.2% prevalence of controlled hypertension and 28.2% prevalence of untreated mild hyperlipidemia—aligns well with the pooled estimates reported by Walters et al. (2025) [12]. The slightly lower prevalence in our study may reflect our exclusion of patients with uncontrolled hypertension or those receiving lipid-lowering therapy, which was necessary to minimize confounding in the assessment of atherogenic indices. Nevertheless, the substantial cardiovascular risk factor burden in our IPF cohort reinforces the need for systematic cardiovascular screening and risk modification strategies in this population.

Several potential pathophysiological mechanisms may underlie the association between IPF and subclinical atherosclerosis observed in our study, though the cross-sectional design precludes causal inference. First, chronic systemic inflammation, a hallmark of IPF, may contribute to endothelial dysfunction and atherogenesis [36,37]. Elevated levels of pro-inflammatory cytokines, such as interleukin-6, tumor necrosis factor-α, and C-reactive protein, have been documented in IPF patients and are known to promote atherosclerotic plaque formation [15,38]. In our study, hs-CRP levels were significantly elevated in IPF patients and correlated independently with CIMT, supporting a possible role for systemic inflammation in vascular remodeling.

Second, oxidative stress, which is implicated in both IPF pathogenesis and atherosclerosis, may represent a shared mechanistic pathway [39,40]. Reactive oxygen species generated during repetitive alveolar injury and aberrant wound healing in IPF may have systemic effects, including oxidation of LDL cholesterol—a critical step in atherogenesis [41]. Third, endothelial dysfunction, characterized by impaired nitric oxide bioavailability and increased expression of adhesion molecules, has been reported in IPF and may predispose to atherosclerotic changes [42,43]. Fourth, chronic hypoxemia, common in advanced IPF, may contribute to cardiovascular dysfunction through activation of hypoxia-inducible factors, increased sympathetic activity, and metabolic derangements [17,18]. However, these proposed mechanisms remain speculative, and longitudinal studies are needed to establish temporal relationships and causality.

The dyslipidemic profile observed in our IPF cohort—characterized by reduced HDL-C and elevated VLDL-C—is consistent with an atherogenic lipid pattern. HDL-C exerts atheroprotective effects through reverse cholesterol transport, antioxidant properties, and anti-inflammatory actions [44]. The reduction in HDL-C observed in IPF patients may therefore contribute to increased cardiovascular risk. Conversely, elevated VLDL-C, a triglyceride-rich lipoprotein, is associated with increased atherosclerotic burden [45]. The mechanisms underlying these lipid alterations in IPF are not fully understood but may involve chronic inflammation, altered hepatic lipid metabolism, and reduced physical activity due to dyspnea and exercise limitation [46,47].

The atherogenic indices evaluated in this study—atherogenic coefficient, CRR, and atherogenic index—integrate information from multiple lipid parameters and have been shown to predict cardiovascular events more accurately than individual lipid measurements in some populations [48,49]. Our finding that all three indices were significantly elevated in IPF patients, and that CRR correlated independently with CIMT, suggests that these simple, cost-effective tools may have utility in cardiovascular risk stratification for IPF patients. However, prospective studies are needed to determine whether these indices predict cardiovascular events or mortality in this population.

A particularly novel finding of our study is the inverse correlation between CIMT and functional capacity (6MWT distance) and the positive correlation between CIMT and dyspnea severity (VAS scores) in IPF patients. These associations suggest potential links between subclinical atherosclerosis and disease-specific functional impairment, though causality cannot be inferred from our cross-sectional data. Several interpretations are possible. First, both CIMT and functional limitation may reflect shared underlying pathophysiological processes, such as chronic inflammation, oxidative stress, and endothelial dysfunction. Second, reduced physical activity due to dyspnea and exercise limitation in IPF may contribute to both cardiovascular deconditioning and atherosclerotic progression [46]. Third, subclinical atherosclerosis may directly impair exercise capacity through reduced cardiac output or peripheral oxygen delivery, though this hypothesis requires further investigation. It is important to note that we did not systematically assess echocardiographic parameters or pulmonary hypertension, which could independently influence exercise capacity and dyspnea and may confound these observed correlations.

The correlations between VAS dyspnea scores and multiple respiratory function parameters (FVC, DLCO) and biochemical markers (hs-CRP) observed in our study are consistent with previous research demonstrating that dyspnea in IPF is multifactorial, reflecting not only impaired gas exchange and lung mechanics but also systemic inflammation and deconditioning [27,50]. The association between dyspnea severity and CIMT suggests that cardiovascular factors may also contribute to symptom burden in IPF, though this hypothesis requires validation in prospective studies with comprehensive cardiovascular phenotyping.

Our subgroup analysis stratified by smoking status demonstrated that the association between IPF and elevated CIMT persisted in both former smokers and never-smokers, suggesting that this relationship is not entirely explained by smoking exposure. However, we acknowledge that the smoking profile differed markedly between IPF patients and controls, with higher former smoking prevalence and greater cumulative pack-year exposure in the IPF group. Despite our subgroup analysis, residual confounding from differential smoking exposure cannot be entirely excluded and represents an important limitation of our study.

Importantly, the adjusted between-group models directly addressed the primary methodological concern regarding confounding. After adjustment for age, sex, cumulative smoking exposure, BMI, and controlled hypertension, IPF status remained independently associated with CIMT and the key atherogenic indices. These findings strengthen the interpretation that the observed vascular differences are associated with IPF status beyond the measured traditional risk factors available in this retrospective dataset. Nevertheless, because of the observational design and the possibility of unmeasured or residual confounding, these results should be interpreted as adjusted associations rather than evidence of causality.

## 5. Clinical Implications

Our findings suggest that systematic cardiovascular risk assessment may be warranted in IPF management. Given the high prevalence of subclinical atherosclerosis and unfavorable atherogenic profiles in IPF patients, clinicians should consider: (1) routine screening for cardiovascular risk factors, including lipid profiles and blood pressure monitoring; (2) non-invasive assessment of subclinical atherosclerosis using CIMT or CAC scoring, particularly in patients with additional cardiovascular risk factors; (3) aggressive management of modifiable cardiovascular risk factors, including smoking cessation, lipid-lowering therapy when indicated, and blood pressure control; and (4) consideration of cardiovascular comorbidities when interpreting functional assessments and planning therapeutic interventions.

Whether early detection and treatment of subclinical atherosclerosis improves outcomes in IPF patients remains unknown and represents an important area for future research. Prospective studies are needed to determine whether cardiovascular risk modification strategies reduce cardiovascular events, improve functional capacity, or enhance survival in this population.

## 6. Limitations

Several limitations of this study should be acknowledged. First, the cross-sectional design precludes causal inference regarding the relationship between IPF and subclinical atherosclerosis. Longitudinal studies with serial CIMT measurements are needed to determine whether atherosclerotic progression is accelerated in IPF and whether it correlates with disease progression or predicts cardiovascular events. Second, the smoking profile differed significantly between IPF patients and controls, with higher former smoking prevalence and greater cumulative pack-year exposure in the IPF group. Although our subgroup analysis stratified by smoking status and the adjusted between-group models demonstrated elevated CIMT in IPF patients after accounting for cumulative smoking exposure, residual confounding from differential smoking exposure cannot be entirely excluded. Third, we did not systematically assess echocardiographic parameters or pulmonary hypertension, which could independently influence exercise capacity, dyspnea burden, and vascular phenotype. The absence of these data limits our ability to fully characterize the cardiovascular phenotype of our cohort and may confound the observed correlations between CIMT and functional parameters. Future studies should include comprehensive echocardiographic evaluation and, where indicated, right heart catheterization to better delineate the interplay between pulmonary vascular disease, systemic atherosclerosis, and functional status in IPF. Fourth, the retrospective design limited our ability to systematically document all comorbidities prospectively. While we enhanced comorbidity documentation in response to reviewer feedback, some relevant clinical information may have been incomplete. Fifth, our study was conducted at a single center, which may limit generalizability to other populations with different demographic characteristics, healthcare systems, or IPF phenotypes. Sixth, we did not assess other markers of subclinical atherosclerosis, such as pulse wave velocity, ankle-brachial index, or coronary artery calcium score, which may provide complementary information. Seventh, we did not evaluate the impact of antifibrotic therapies (nintedanib, pirfenidone) on CIMT or atherogenic indices, as many patients in our cohort were not receiving these medications at the time of enrollment. Future studies should examine whether antifibrotic therapies influence cardiovascular risk profiles in IPF. Finally, the relatively modest sample size may have limited statistical power to detect smaller effect sizes or to perform more complex multivariable analyses.

## 7. Conclusions

This study demonstrates that patients with IPF have higher CIMT and more unfavorable atherogenic profiles compared with healthy controls. These differences persisted after adjustment for age, sex, smoking exposure, BMI, and controlled hypertension, suggesting an independent association between IPF status and markers of subclinical atherosclerosis. The inverse association between CIMT and functional capacity, together with the positive association between CIMT and dyspnea severity, indicates a potential relationship between vascular remodeling and disease-specific functional impairment. However, causality cannot be inferred from the present cross-sectional data. These findings support the integration of systematic cardiovascular risk assessment into IPF management and highlight the need for prospective studies with comprehensive cardiovascular phenotyping, including echocardiography and pulmonary hypertension assessment.

## Figures and Tables

**Table 1:** Baseline Demographic, Clinical, and Laboratory Characteristics.

Characteristic	IPF Patients (*n* = 117)	Controls (*n* = 104)	*p*-Value
Age (years), mean ± SD	66.4 ± 8.7	65.8 ± 8.3	0.612
Male sex, *n* (%)	84 (71.8)	72 (69.2)	0.673
BMI (kg/m^2^), mean ± SD	26.8 ± 4.2	28.1 ± 3.9	0.021
**Smoking Status, *n* (%)**			
Never smoker	32 (27.4)	58 (55.8)	<0.001
Former smoker	73 (62.4)	30 (28.8)	<0.001
Current smoker	12 (10.3)	16 (15.4)	0.244
Pack-years, mean ± SD	28.6 ± 22.4	12.3 ± 15.7	<0.001
**Comorbidities, *n* (%)**			
Controlled hypertension	40 (34.2)	19 (18.3)	0.007
Untreated mild hyperlipidemia	33 (28.2)	22 (21.2)	0.224
Other vascular disorders	7 (6.0)	2 (1.9)	0.134
**Laboratory Parameters**			
Fasting glucose (mg/dL), mean ± SD	94.6 ± 11.2	92.8 ± 10.4	0.223
Creatinine (mg/dL), mean ± SD	0.89 ± 0.18	0.87 ± 0.16	0.412
hs-CRP (mg/L), median (IQR)	3.8 (2.1–6.4)	1.9 (1.2–3.2)	<0.001

BMI, body mass index; hs-CRP, high-sensitivity C-reactive protein; IQR, interquartile range; SD, standard deviation.

**Table 2:** Pulmonary Function and Functional Capacity Parameters.

Parameter	IPF Patients (*n* = 117)	Controls (*n* = 104)	*p*-Value
FVC (% predicted), mean ± SD	62.4 ± 14.8	98.6 ± 12.3	<0.001
FEV_1_ (% predicted), mean ± SD	64.8 ± 15.2	96.4 ± 13.1	<0.001
FEV_1_/FVC ratio, mean ± SD	0.82 ± 0.06	0.79 ± 0.05	0.001
DLCO (% predicted), mean ± SD	48.7 ± 13.6	94.2 ± 11.8	<0.001
6MWT distance (m), mean ± SD	348.6 ± 92.4	486.3 ± 68.7	<0.001
VAS dyspnea score (mm), mean ± SD	54.3 ± 18.6	8.2 ± 6.4	<0.001

DLCO, diffusing capacity for carbon monoxide; FEV_1_, forced expiratory volume in 1 s; FVC, forced vital capacity; 6MWT, 6-min walk test; SD, standard deviation; VAS, visual analog scale.

**Table 3:** Lipid Profiles and Atherogenic Indices.

Parameter	IPF Patients (*n* = 117)	Controls (*n* = 104)	*p*-Value
Total cholesterol (mg/dL), mean ± SD	198.4 ± 38.6	192.3 ± 36.2	0.234
Triglycerides (mg/dL), mean ± SD	142.1 ± 48.2	120.6 ± 39.1	0.001
HDL-C (mg/dL), mean ± SD	46.3 ± 11.2	52.1 ± 10.8	<0.001
LDL-C (mg/dL), mean ± SD	123.7 ± 32.4	116.1 ± 30.8	0.082
VLDL-C (mg/dL), mean ± SD	28.4 ± 9.6	24.1 ± 7.8	0.001
**Atherogenic Indices**			
Atherogenic coefficient, mean ± SD	3.68 ± 1.18	3.12 ± 0.94	<0.001
Cholesterol ratio risk, mean ± SD	4.12 ± 1.23	3.45 ± 0.98	<0.001
Atherogenic index, mean ± SD	0.54 ± 0.22	0.43 ± 0.18	<0.001

HDL-C, high-density lipoprotein cholesterol; LDL-C, low-density lipoprotein cholesterol; SD, standard deviation; VLDL-C, very-low-density lipoprotein cholesterol.

**Table 4:** Carotid Intima–Media Thickness and Plaque Prevalence.

Parameter	IPF Patients (*n* = 117)	Controls (*n* = 104)	*p*-Value
CIMT (mm), mean ± SD	0.78 ± 0.12	0.68 ± 0.10	<0.001
Abnormal CIMT (>0.9 mm), *n* (%)	34 (29.1)	10 (9.6)	<0.001
Presence of plaques, *n* (%)	28 (23.9)	12 (11.5)	0.015

CIMT, carotid intima–media thickness; SD, standard deviation.

**Table 5:** Correlations Between CIMT and Clinical Parameters in IPF Patients.

Parameter	Correlation with CIMT	
	r	*p*-Value
Age	0.412	<0.001
BMI	0.156	0.094
Pack-years	0.234	0.012
FVC (% predicted)	−0.246	0.008
DLCO (% predicted)	−0.268	0.004
6MWT distance	−0.312	0.001
VAS dyspnea score	0.287	0.002
Total cholesterol	0.198	0.033
HDL-C	−0.276	0.003
Atherogenic coefficient	0.318	<0.001
Cholesterol ratio risk	0.342	<0.001
Atherogenic index	0.294	0.001
hs-CRP	0.324	<0.001

BMI, body mass index; CIMT, carotid intima–media thickness; DLCO, diffusing capacity for carbon monoxide; FVC, forced vital capacity; HDL-C, high-density lipoprotein cholesterol; hs-CRP, high-sensitivity C-reactive protein; 6MWT, 6-min walk test; VAS, visual analog scale.

**Table 6:** CIMT Stratified by Smoking Status.

Smoking Status	IPF Patients	Controls	*p*-Value
**Former Smokers**			
*n*	73	30	
CIMT (mm), mean ± SD	0.80 ± 0.13	0.72 ± 0.11	0.006
**Never-Smokers**			
*n*	32	58	
CIMT (mm), mean ± SD	0.74 ± 0.10	0.66 ± 0.09	0.002

CIMT, carotid intima–media thickness; SD, standard deviation.

**Table 7:** Adjusted Between-Group Comparisons for CIMT and Key Atherogenic Indices.

Outcome	Main Predictor	Adjusted β	95% CI	*p*-Value
CIMT, mm	IPF vs. control	0.086	0.057 to 0.115	<0.001
Cholesterol ratio risk	IPF vs. control	0.482	0.191 to 0.773	0.001
Atherogenic coefficient	IPF vs. control	0.481	0.190 to 0.772	0.001
Atherogenic index	IPF vs. control	0.079	0.026 to 0.132	0.004

β coefficients represent adjusted mean differences for IPF patients compared with controls (reference group). Smoking exposure was handled as cumulative pack-years in the primary adjusted model. AC, atherogenic coefficient; AI, atherogenic index; BMI, body mass index; CI, confidence interval; CIMT, carotid intima–media thickness; CRR, cholesterol ratio risk; IPF, idiopathic pulmonary fibrosis.

**Table 8:** Multiple Linear Regression Analysis: Independent Predictors of CIMT in IPF Patients.

Variable	Unstandardized β	Standard Error	Standardized β	*p*-Value
Age (years)	0.004	0.001	0.312	<0.001
Male sex	0.018	0.022	0.068	0.412
Pack-years	0.002	0.001	0.186	0.018
BMI (kg/m^2^)	0.001	0.002	0.042	0.624
FVC (% predicted)	−0.001	0.001	−0.098	0.234
Cholesterol ratio risk	0.012	0.004	0.246	0.006
hs-CRP (mg/L)	0.008	0.003	0.212	0.012

Adjusted R^2^ = 0.483; *p* < 0.001 for overall model. BMI, body mass index; CIMT, carotid intima–media thickness; FVC, forced vital capacity; hs-CRP, high-sensitivity C-reactive protein.

## Data Availability

The datasets used and analyzed during the current study are available from the corresponding author on reasonable request.

## Abbreviations

6MWT: 6-min walk test; AC: atherogenic coefficient; AI: atherogenic index; ATS: American Thoracic Society; BMI: body mass index; CAC: coronary artery calcium; CCA: common carotid artery; CIMT: carotid intima–media thickness; CRR: cholesterol ratio risk; CT: computed tomography; DLCO: diffusing capacity for carbon monoxide; ERS: European Respiratory Society; FEV_1_: forced expiratory volume in 1 s; FVC: forced vital capacity; HDL-C: high-density lipoprotein cholesterol; HRCT: high-resolution computed tomography; hs-CRP: high-sensitivity C-reactive protein; IPF: idiopathic pulmonary fibrosis; IQR: interquartile range; LDL-C: low-density lipoprotein cholesterol; SD: standard deviation; UIP: usual interstitial pneumonia; VAS: visual analog scale; VLDL-C: very-low-density lipoprotein cholesterol.
